# Identification and immune characteristics of N6-methyladenosine related ferroptosis-related genes in intervertebral disc degeneration

**DOI:** 10.1038/s41598-025-18011-z

**Published:** 2025-09-29

**Authors:** Zhen Che, Ruibing Chen, Ming Li, Zhuangyao Liao, Kun Wang, Dengbo Yao, Yuwei Liang, Yuxi Li, Guoming Wen, Tong Xing, Kaihui Su, Changchun Liang, Lin Huang, Qun Zhao

**Affiliations:** 1 Experimental Orthopedics and Trauma Surgery, University Hospital RWTH Aachen, Pauwelsstraße 30, 52074 Aachen, Germany; 2 Stomatological College of Nanjing Medical University, Jiangsu, 211166 China; 3 Department of Orthopedics, Sun Yat-Sen Memorial Hospital, Sun Yat-Sen University (SYSU), Guangzhou, 510120 China

**Keywords:** Intervertebral disc degeneration, N6-methyladenosine, Ferroptosis, Immune, Molecular biology, Biomarkers, Diseases, Medical research, Molecular medicine

## Abstract

The damage of ferroptosis is related to the pathogenesis of intervertebral disc degeneration (IDD). N6-methyladenosine (m6A) modification accounts for more than 80% of RNA modifications in eukaryotic cells. However, the key role of m6A related ferroptosis-related genes (FRGs) in IDD remain explorable. Firstly, batch correction between datasets was performed. Weighted gene co-expression network analysis (WGCNA) was then conducted to acquire the most relevant module genes. Differentially expressed genes (DEGs) between the IDD and Normal Group were obtained by differential analysis subsequently. Next, according to the Pearson correlation and the intersection of m6A and FRGs related genes, m6A-FRGs related genes were selected. Additionally, we intersected module genes, DEGs and m6A-FRGs related genes to obtain DEGs of m6A-FRGs related module genes (m6A-FRGs-DEGs). Least absolute shrinkage and selection operator (LASSO) was applied to get HUBgenes. After, we conducted gene set enrichment analysis (GSEA) to gain function items and related pathways of HUBgenes. Single sample gene set enrichment analysis (ssGSEA) and wilcox.test were proceeded to analyse differences in relative abundance of immune cells between the IDD and Normal Groups, and a nomogram was constructed based on significantly different HUBgenes. The Comparative Toxicogenomics Database (CTDbase) was then applied to predict potential drugs or molecular compounds that could modulate HUBgenes. Last but not least, we performed the quantitative real-time fluorescence PCR (qRT-PCR) to verify HUBgenes. 181 m6A-FRGs-DEGs were acquired by the intersection of key module genes (from MEtan, MEsalmon, MEbrown and MEgreen), 362 DEGs and 15,678 m6A-FRGs related genes. Subsequent analysis showed that HUBgenes (ZNF595, PLXDC1, FNBP1L, KLRB1, NRCAM, PPCDC, C9orf139, SIGLEC17P, RRAS2 and DPRXP4) significantly participated in positive regulation of cytokine production, mitochondrial inner membrane and organellar ribosome. Besides, the relative abundance of neutrophils was found significantly different between IDD and normal groups. A nomogram was constructed based on ZNF595 and RRAS2, and there were 11 drugs targeted on ZNF595, while 118 drugs predicted based on RRAS2 such as Tetrachlorodibenzodioxin, Bisphenol A and Benzo(a)pyrene. Lastly, ZNF595 and RRAS2 were both obviously up-regulated in IDD according to the qRT-PCR. Our research suggested 10 HUBgenes were significantly associated with IDD, providing more evidence about the vital role of HUBgenes in IDD.

**Supplementary Information:**

The online version contains supplementary material available at 10.1038/s41598-025-18011-z.

## Introduction

Intervertebral disc degeneration (IDD) is one of the most common degenerative diseases of musculoskeletal disorders (MSKs)^1^. It can lead to low back pain (LBP), a primary and universal cause of disability^2^. The incidence of IDD is mostly concentrated in middle-aged and elderly people, which increases in prevalence with age^3^. However, research implicated that early IDD in the young was nonnegligible as well^4,5^. Therefore, IDD not only seriously affects the quality of patients’ life but also contributes to huge social and economic burden^6^.

IDD results from a lot of factors, which include genetic inheritance, ever-increasing aging, mechanical injury, malnutrition, etc^7^. Pathological changes of intervertebral disc (IVD) mainly consist of the narrowing of intervertebral space, dysfunction of nucleus pulposus cell (NPC) like senescence and apoptosis, progressive degradation of extracellular matrix (ECM), rupture and fibrosis of annulus fibrosus (AF), calcification of cartilaginous endplates (CEPs) and so on^8^. However, although scientists have already made a lot of effort, the exact etiology and molecular mechanism of IDD remain unclear, which are still worth exploring in the foreseeable future. Meanwhile, as tissue engineering^9^, growth factor therapy^10^, gene therapy^11^, small molecule-based treatment approach^12^, and cell-based treatment^13^ are proposed to regenerate IVD, it is still a fully noteworthy direction to explore new therapies aiming at diverse targets for IDD.

Nowadays, ferroptosis, one of the forms of programmed cell death (PCD), has been increasingly attractive as a conceivable therapeutic target for IDD^14–16^. It is driven by iron-dependent phospholipid peroxidation and regulated by multiple cellular metabolic pathways, including redox homeostasis, iron handling, mitochondrial activity and metabolism of amino acids, lipids and sugars, in addition to various signaling pathways relevant to degenerative diseases^17,18^. As such, pharmacological modulation of ferroptosis, via both its induction and its inhibition, holds great potential for the treatment of IDD linked to extensive lipid peroxidation^19^. Several studies have shown that the pathogenesis of IDD involved ferritin degradation mediated by ferritin phagocytosis and subsequent lipid peroxidation. In a rat model of IDD, the levels of GPX4, an inhibitor of ferroptosis, and ferritin heavy chain were found significantly lower than the control group^20,21^. In addition, while injury-induced IDD process could be attenuated by deferoxamine, another inhibitor of ferroptosis^20^, it can be promoted by iron overload through inducing oxidative stress and ferroptosis in endplate chondrocytes^22^. Thus, the inhibition of NPC ferroptosis has been already suggested as a probable effective strategy to alleviate IDD^23^. Wenkai et al. (2025) found that through marked ferroptosis inhibition in NPCs that not only prevents LDH recurrence but also reverses the IDD symptoms, leading to robust restoration of NP structure and functions^24^. Jitian et al. (2025) also suggested that ferroptosis was identified as critical molecular entities and cellular pathways implicated in reactive oxygen species (ROS)-mediated IDD^25^. Therefore, studying the role of ferroptosis in IDD will help us to reveal the molecular mechanism of IDD to a higher degree and provide further references for therapeutic strategies.

Epigenetic modification means that gene expression and function change without changing the gene sequence, including chemical modifications of DNA, RNA and proteins. For instance, Yongzhao et al. (2025) constructed a novel nanoparticle named Hi-Exos to reduce the senescence of NPC and repair IDD via epigenetic modification, involving the epigenetic factor miR-221-3p^26^. Shibin et al. (2025) also found that the activator protein-1 (AP-1) transcription factor critical in driving the chromatin accessibility changes during IDD^27^. Among many epigenetic modifications of inflammation in IDD^28^, N6-methyladenosine (m6A) methylation is not only the most abundant internal modification of mRNA in eukaryotes, but also is widely discovered in non-coding RNAs, such as rRNAs and snRNAs^29–31^. Briefly said, m6A methylation transfers methyl to the N-6 position of adenosine in RNA^32^. It has a conserved modified gene sequence, which is enriched in long exons, near stop codons and 3’-untranslated regions (3’-UTR)^33,34^. Moreover, the m6A methylation process is dynamic and reversible, regulated by “writers”, “erasers” and “readers”^35^. Some researchers have elucidated the close relationship between m6A and ferroptosis, indicating that targeting m6A to influence ferroptosis might be a promising therapy for IDD^36–38^. Nevertheless, the key role of m6A related ferroptosis-related genes (FRGs) in IDD still remains unreported.

Therefore, in this study, we applied bioinformatic methods to identify m6A related FRGs and suggested HUBgenes, which were significantly related to IDD. Through a series of analyses, the study offered more evidence about the vital role of HUBgenes in IDD. In this way, we hoped to elucidate the molecular mechanism that underlies IDD, leading us to identify receptors and bridging molecules of m6A and ferroptosis, which can be useful in incidence or severity prediction of IDD and guide better health care for patients.

## Materials and methods

### Date sources and reduction

There were 17 normal and 17 IDD blood samples in transcriptome dataset GSE150408 obtained from the Gene Expression Omnibus (GEO) (https://www.ncbi.nlm.nih.gov/geo/). The transcriptome dataset GSE124272 has 8 normal and 8 IDD blood samples, as GSE167199 has 3 normal and 3 IDD nucleus pulposus samples. 27 m6A methylation regulatory factors were acquired from the research conducted by Lu, et al^39^. There were 9 FRGs in the ferroptosis database (FerrDb V2) (http://www.zhounan.org/ferrdb). Principal component analysis (PCA) was used to batch effect between GSE150408 and GSE124272 datasets. Batch correction between IDD and normal groups was performed by the limma package (version 3.44.3), and the batch effect between datasets were tested by PCA again^40^.

### Weighted gene co-expression network analysis (WGCNA)

The WGCNA algorithm was applied to construct and analyze the co-expression network of the whole gene expression profiles of normal and IDD samples in the merged dataset. The IDD and normal samples were used as WGCNA trait data to screen the highest correlation modules and genes related to IDD. Samples of the merged dataset (25 IDD and 25 normal samples) were used to construct a co-expression network by WGCNA package^41^. The overall correlation of all samples in the dataset was obtained by clustering, with Z.k < −2.5 as the connectivity screening threshold, and outlier samples were eliminated to ensure the accuracy of the analysis. The soft threshold on the data was determined to ensure that the intergenic interaction between genes conforms to the scale-free distribution to the maximum extent. Adjacency and similarity between genes were calculated to derive the dissimilarity coefficient, then the system clustering tree among genes was obtained. According to the standard of mixed dynamic shear tree, the minimum number of genes of per gene module were set to 50. MEDissThres were set to 0.3 to merge similar modules. The correlation analyses of gene modules with IDD and normal samples were performed, we transformed phenotypic traits into quantitative traits, with |Cor|> 3 and p < 0.01 were set as the threshold to screen modules.

### Differential expression analysis and correlation analysis

Limma package was applied to obtain the differentially expressed genes (DEGs) between the normal and IDD groups in the merged dataset (|log_2_FC|> 0.5, p < 0.05). The ggplot2 package (version 3.3.2) and the heatmap package (version 0.7.7) were used to plot the volcano and heatmap, respectively^42^. Pearson correlations between 27 m6A methylation regulators and all genes in the merged dataset were calculated, and correlations between 9 FRGs and all genes in the merged dataset were calculated by pearson (|Cor|> 0.3 and p < 0.01). m6A-FRGs related genes were obtained by the intersection of m6A related and FRGs related genes.

### Differential expression module gene

Module genes, DEGs and m6A-FRGs related genes were intersected to obtain m6A-FRGs related DEGs (m6A-FRGs-DEGs). Gene ontology (GO) and Kyoto Encyclopedia of Genes and Genomes (KEGG) enrichment analysis of m6A-FRGs-DEGs were performed by clusterProfiler (version 3.2–3)^43–46^. The STRING online database (http://cn.string-db.org) was applied to construct a protein–protein interaction (PPI) network of m6A-FRGs-DEGs^47^. The results were visualized by Cytoscape v.3.9.1 and used for constructing the enrichment analysis pathway network of m6A-FRGs-DEGs by the clueGO plug-in (p < 0.05).

### Screening of HUBgenes

HUBgenes of IDD were screened by least absolute shrinkage and selection operator (LASSO) to further evaluate the value of m6A-FRGs-DEGs^48^. We performed 5 types of machine learning methods on HUBgenes, including Logistic Regression (LR), Random Forest (RF), eXtreme Gradient Boosting (XGBoost), Adaptive Boosting (AdaBoost), and Support Vector Machine (SVM)^49–53^. The receiver operating characteristic (ROC) curves of 5 types of machine learning were drawn by the survival ROC to verify the credibility of HUBgenes^54^.

### Enrichment analysis of HUBgenes

GOSemSim package was employed to perform functional similarity (friend) analysis on HUBgenes^55^. Functionally similar significantly correlated HUBgenes were visualized by boxplots. We calculated the association of HUBgenes with other genes in the merged dataset, and according to the correlation ranking, Gene Set Enrichment Analysis (GSEA) was performed on HUBgenes by clusterProfiler (version 4.0.2) and org.Hs.eg.db (version 3.13.0)^43^. GO and KEGG gene sets were applied to assess enrichment analysis of related pathways and molecular mechanisms of IDD, the minimum and maximum gene set sizes were set as 10 and 500, respectively (p < 0.05 and q < 0.25)^44–46^.

### Immune infiltration and cytokine analysis

In the study, we used 29 immune-related gene sets (including 16 gene sets of immune cells and 13 immune-related pathways), including immune cell species, immune-related pathways and functions^56^. In order to further explore the infiltration of immune cells in normal and IDD samples, we analyzed the relative abundance of immune cells in GSE124272 and GSE150408. The single sample GSEA (ssGSEA) algorithm was applied to estimate the relative abundance of 29 immune cells. Differences in relative abundance of immune cells between normal and IDD samples were calculated by wilcox.test, and the immune cells with significant differences in both datasets would be selected as the key differential immune cells. ROC curves were plotted to determine the credibility of key differential immune cells. The correlation between HUBgenes and differential immune cells in the two datasets was calculated, respectively. And correlation network map was drawn (|Cor|> 0.3 and p < 0.05). Studies have already shown that NPC degeneration could increase the secretion of proinflammatory cytokines^23^. Therefore, the pearson correlation between HUBgenes and common cytokines in the merged dataset was analyzed. And significant correlation between HUBgenes and common cytokines were visualized by the heatmap.

### Relationship between HUBgenes and clinical features, and the identification of potential drugs

In order to verify the effectiveness of the selected genes, the expression of HUBgenes were verified in GSE167199. And the correlation between HUBgenes obtained by verification (p < 0.05) and clinical features in GSE167199 was calculated. Next, SVM prediction model and ROC curve were constructed for HUBgenes verificated. A nomogram was constructed based on HUBgenes that were significantly different in the validation set, and the calibration curve was drawn based on the prediction model. Then, we applied the Comparative Toxicogenomics Database (CTDbase) (http://ctdbase.org/) to predict potential drugs or molecular compounds that could modulate HUBgenes that were significantly different in the validation set. And genes-drugs regulatory network was drawn based on all drugs and HUBgenes.

### Experimental verification

All samples were obtained from IDD patients with knowledge and consent from Sun Yat-sen Memorial Hospital, Sun Yat-sen University (SYSU). The study has already been approved by the Sun Yat-sen Memorial Hospital Medical Ethics Committee. 20 frozen tissue samples of IVDs were divided into two groups, of which 10 normal and 10 IDD samples. Firstly, total RNA of samples was isolated and purified by TRIzol (Ambion) reagent following the instruction manual. Then, the extracted RNA was tested for concentration by NanoPhotometer N50. Next, reverse transcription via SureScript-First-strand-cDNA-synthesis-kit (Servicebio) by an ordinary PCR instrument. Reverse transcription product cDNA was diluted 5 to 20 times with ddH2O (RNase/ DNase free). Subsequently, polymerase chain reaction (PCR) amplification reaction was performed by CFX96 real-time quantitative PCR instrument. 1 min at 95 ℃ (pre-denaturation), followed by at 95 ℃ for 20 s (denaturation), 55 ℃ for 20 s (annealing) and 72 ℃ for 30 s (elongation). The above reactions were subjected to forty cycles. Primer sequences were shown in Table 1. The quantitative real-time fluorescence PCR (qRT-PCR) was performed to verify the expression levels of HUBgenes that were significantly different in the validation set in IDD.

**Table 1:** Primer sequences.

Primer	Sequence
RRAS2 F	CCGCTTCAAGTACTGTGTATTTCTT
RRAS2 R	CGGGCTGCTCTGTCATCTAT
ZNF595 F	CTGCGAGGGCTTGGTTTAGG
ZNF595 R	GGAGAACATATAGCATTTCCCGAC
internal reference-GAPDH F	CGAAGGTGGAGTCAACGGATTT
internal reference-GAPDH R	ATGGGTGGAATCATATTGGAAC

### Statement

All methods in the present article were performed in accordance with the relevant guidelines and regulations. Also, as for the involving human tissue samples used in the study, informed consent was obtained from all subjects and/or their legal guardian(s).

## Results

### Data consolidation and batch correction

**Fig. 1:**
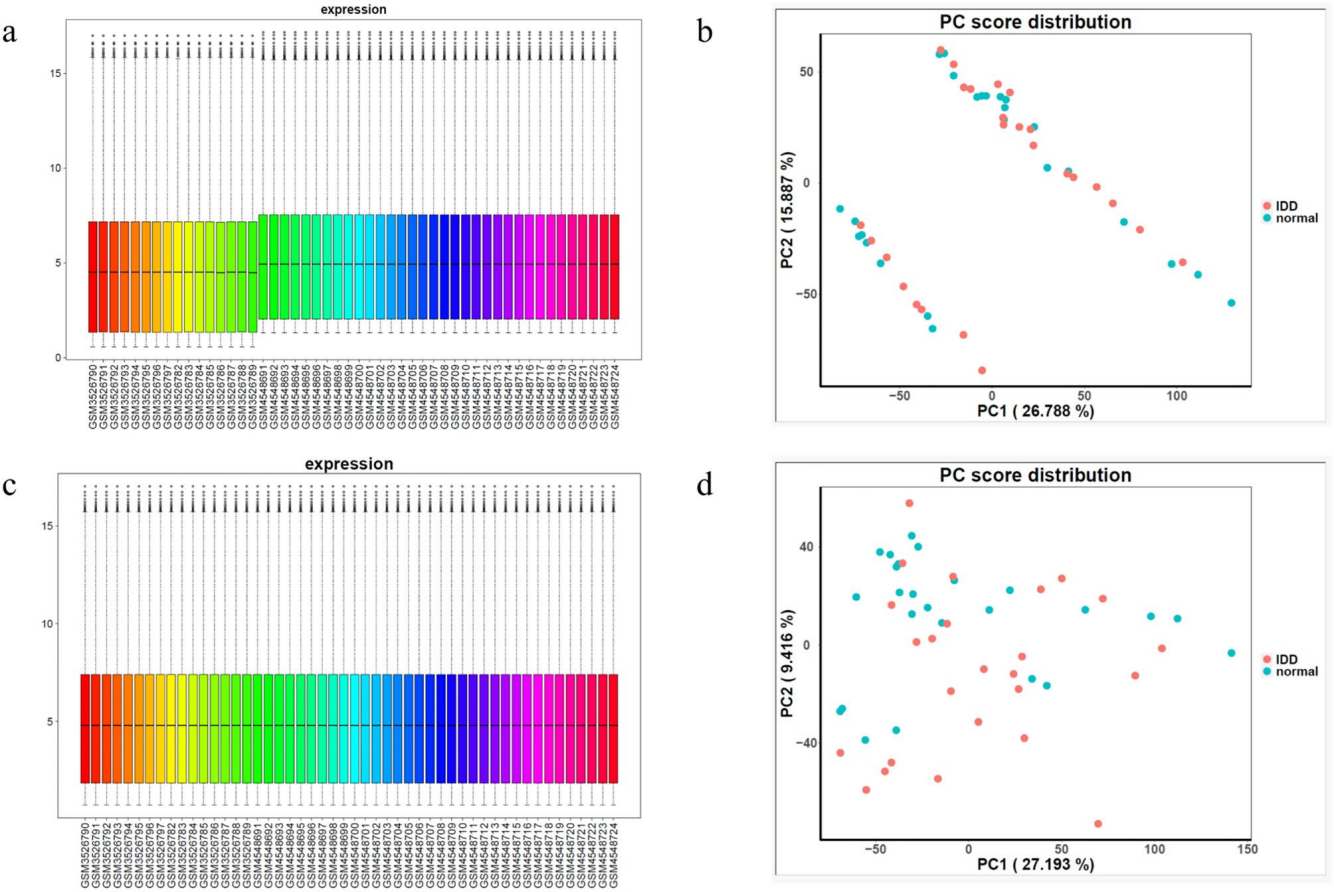
Dataset batch correction: (**a**) Dataset expression box plot before batch correction; (**b**) Dataset PCA main component scatter plot before batch correction; (**c**) Dataset expression box plot after batch correction; (**d**) PCA main component scatter plot after batch correction.

There were significant systematic differences in gene expression between GSE150408 and GSE124272 before batch correction (Fig. 1a). PCA showed that samples were obviously divided into two clusters (Fig. 1b). After batch correction, the system differences between datasets GSE150408 and GSE124272 were eliminated (Fig. 1c). PCA showed that the samples clearly have been fused into one cluster (Fig. 1d). It was indicated that two datasets have been effectively merged, and there was no systematic error in the merged new dataset for subsequent analysis.

### Screening module genes by WGCNA

**Fig. 2:**
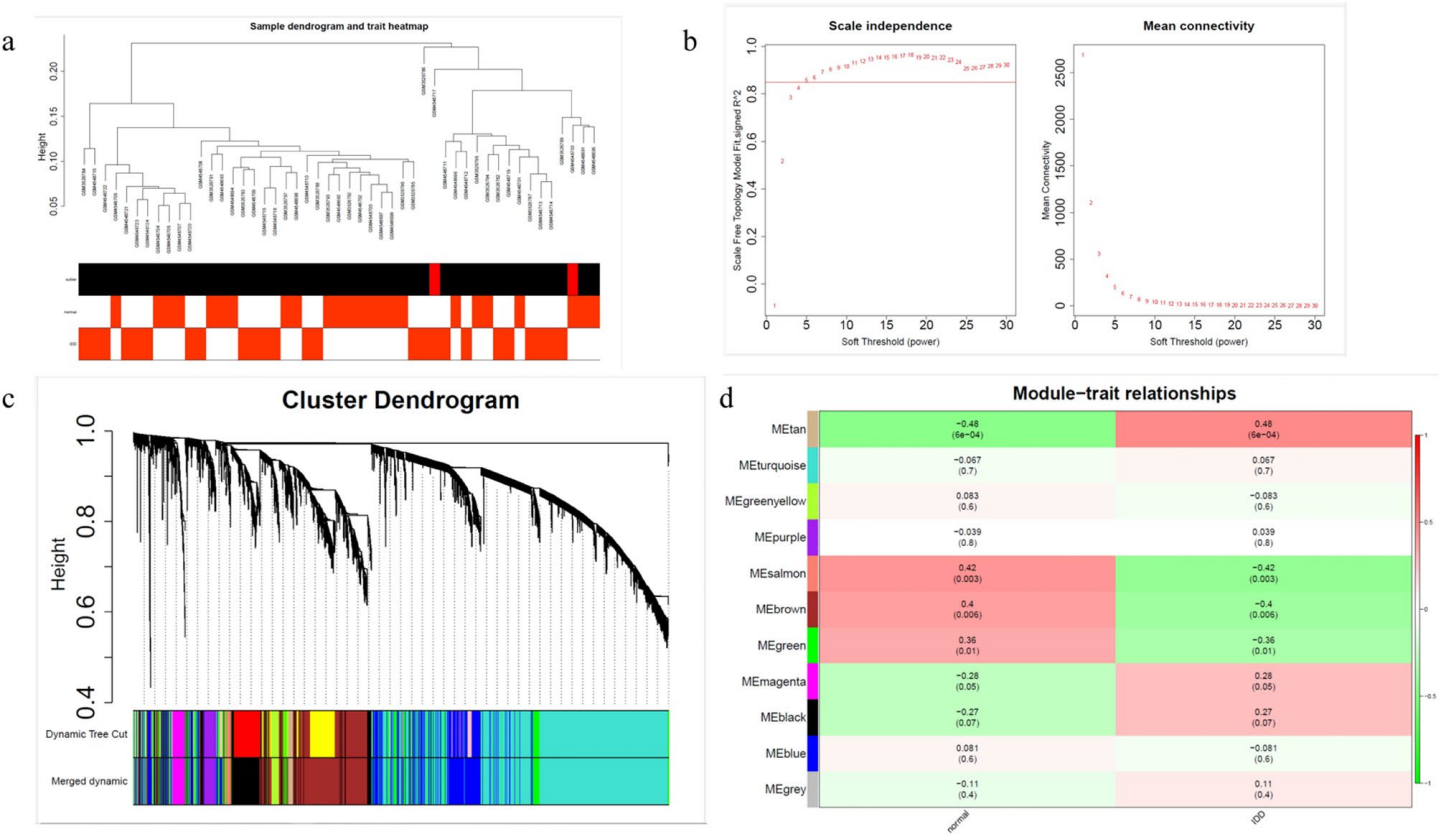
(**a**) Sample clustering tree; (**b**) Scale-free soft threshold distribution. The higher the square of the correlation coefficient, the closer the network is to the distribution without network scale; (**c**) Module merging. Genes are divided into various modules through hierarchical clustering. Different colors represent different modules. Gray is the default gene that cannot be classified into any module; (**d**) Heat map of module and trait correlation. Red in the figure represents positive correlation, green represents negative correlation, and the color depth represents the degree of correlation.

Sample clustering result showed that GSM4548717 and GSM3526786 among all samples were outliers (Fig. 2a). The soft threshold was determined as 5, so that the network was closest to the distribution without network scale (Fig. 2b). 14 modules were obtained by mixed dynamic shear tree, and there were 11 modules left after merging (Fig. 2c). Among the 11 co-expression modules, MEtan has the highest correlation with normal and IDD phenotypic traits (normal: Cor = -0.48 and p = 6e-04, IDD: Cor = 0.48 and p = 6e-04), with a negative correlation with normal while a positive correlation with IDD. In addition, MEsalmon (|Cor|= 0.42, p = 0.003), MEbrown (|Cor|= 0.4, p = 0.006) and MEgreen (|Cor|= 0.36, p = 0.01) were also highly correlated with phenotypic traits. Therefore, MEtan, MEsalmon, MEbrown and MEgreen modules were selected as candidate key modules (2749 genes) for subsequent analyses (Fig. 2d).

### Differential expression analysis and correlation analysis between normal and IDD group

**Fig. 3:**
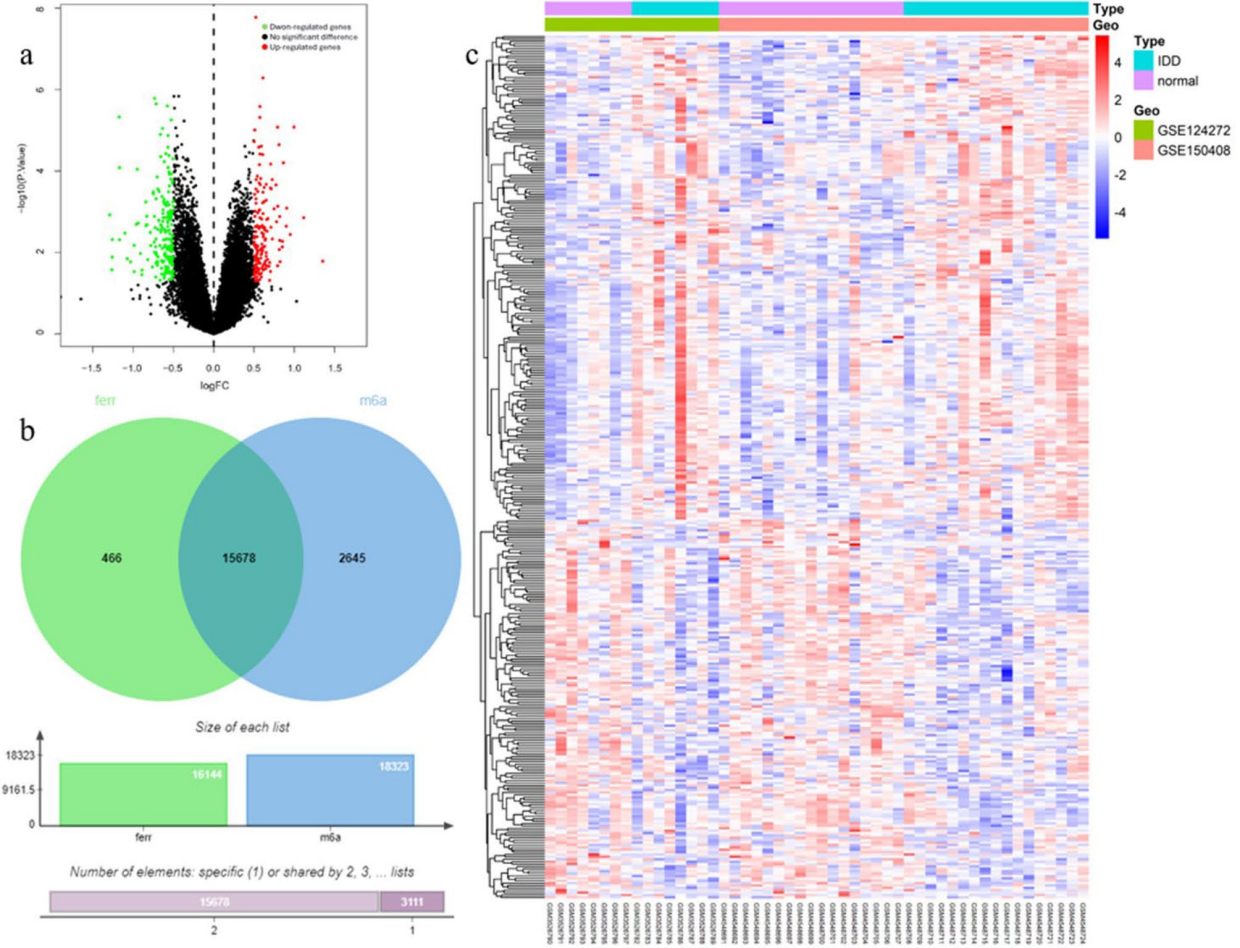
(**a**, **b**) Volcano plot and heat map of differentially expressed genes between sample groups: (**a**) Volcano plot of differential genes. Each point in the figure represents a gene, green and red points represent significantly differentially expressed genes. The red dots indicate that the gene expression level is up-regulated, the green indicate down-regulated, and the black indicate that there is no significant difference between these genes; (**b**) Differential gene heat map. Each square in the heat map represents the relative expression of a gene in different samples. Red indicates high relative expression and blue indicates low; (**c**) The intersection and correlation analysis of m6A-FRGs related genes.

There were 362 DEGs between normal and IDD groups, of which 159 were up-regulation and 203 were down-regulation (Fig. 3a). Heat map showed the expression of DEGs between IDD and Normal group (Fig. 3b). Then, 15,678 m6A-FRGs related genes were obtained by intersection and correlation analysis (Fig. 3c).

### Enrichment analysis and construction of m6A-FRGs-DEGs

**Fig. 4:**
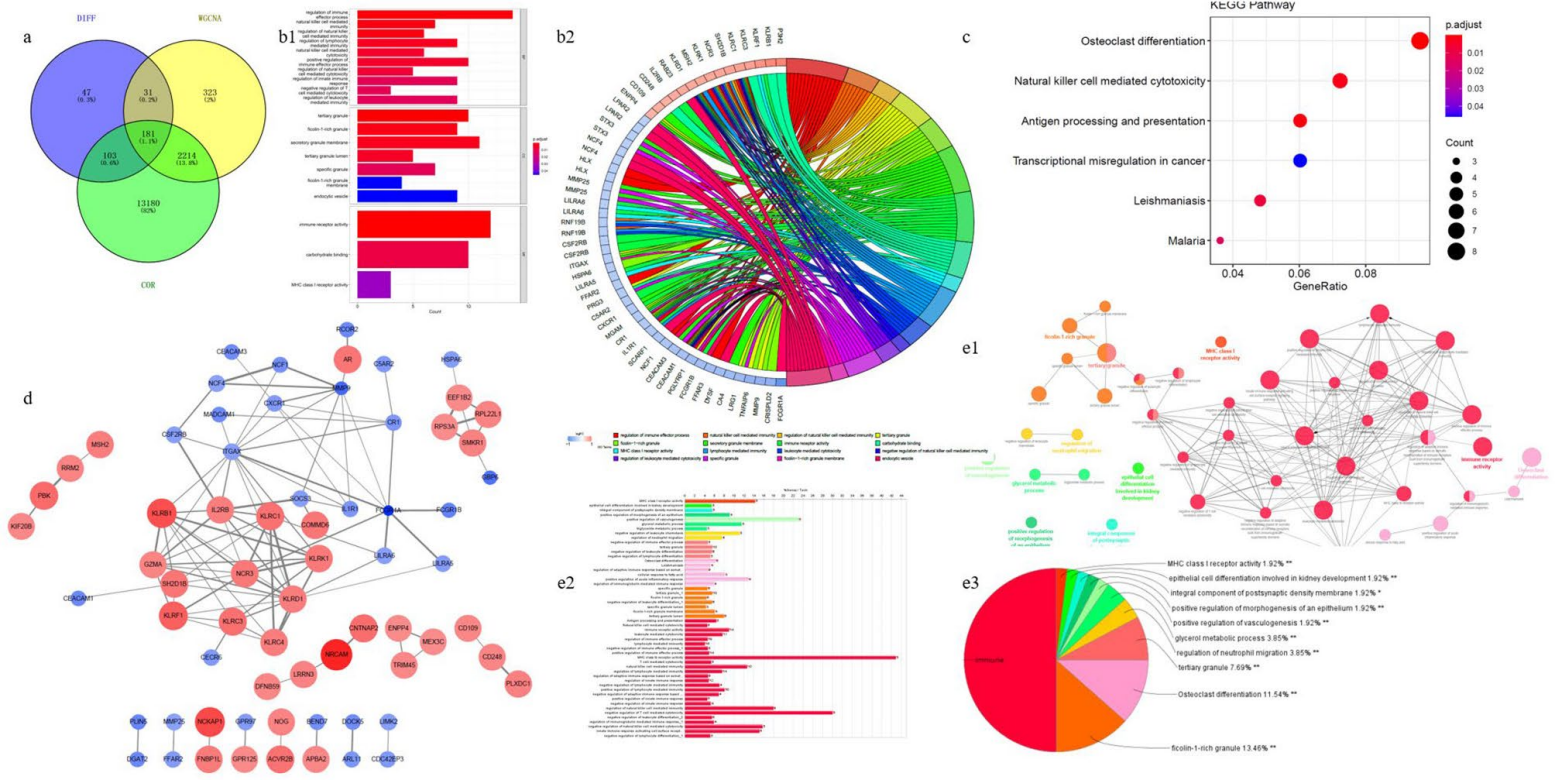
(**a**) DE-COR-ME Venn. The blue circle represents DEGs, the yellow represents MEgenes, and the green represents the COR gene set; (**b**) GO enrichment results of differentially expressed genes: (**b1**) GO enrichment bar chart (the top 10 were screened and drawn according to significance ranking). The color represents p value. The redder the color, the smaller the value; (**b2**) GO enrichment chord diagram (top 16 were filtered for visualization based on significance ranking); (**c**) KEGG functional enrichment analysis; (**d**) PPI of m6A-FRGs-DEGs. Red nodes are up-regulated proteins, blue nodes are down-regulated, color depth and node size are distributed according to logFC, and the thickness of the connecting line is sorted according to the confidence of protein interaction; (**e**) Enrichment network analysis of m6A-FRGs-DEGs: (**e1**) The node colors represent different enriched pathway categories, the color ratio represents the number of genes overlapped by each pathway, and the thickness of the connecting line represents the degree of association between pathways; (**e2**) The bar graph corresponding to the enriched network. The length of the bar graph represents the number of enriched genes; (**e3**) The pie chart corresponding to the network diagram, the area of the pie chart represents the proportion of enriched pathway categories.

There were 181 m6A-FRGs-DEGs obtained by the intersection (Fig. 4a). In the GO functional enrichment analysis, m6A-FRGs-DEGs were mainly enriched in regulation of immune effector process, natural killer (NK) cell mediated immunity and immune receptor activity (Fig. 4b, Supplementary Data). Then in the KEGG functional enrichment analysis, m6A-FRGs-DEGs were involved in osteoclast differentiation, NK cell mediated cytotoxicity, antigen processing, and presentation (Fig. 4c). Notably, there were 12 m6A-FRGs-DEGs (KLRB1, IL2RB, KLRC1, COMMD6, GZMA, SH2D1B, NCR3, KLRD1, KLRK1, KLRF1, KLRC3 and KLRC4) in the PPI network (Fig. 4d). Moreover, enrichment analysis pathway network showed that m6A-FRGs-DEGs were mainly participated in ficolin-1-rich granule, osteoclast differentiation and immune receptor activity (Fig. 4e).

### Screening HUBgenes by machine learning methods

**Fig. 5:**
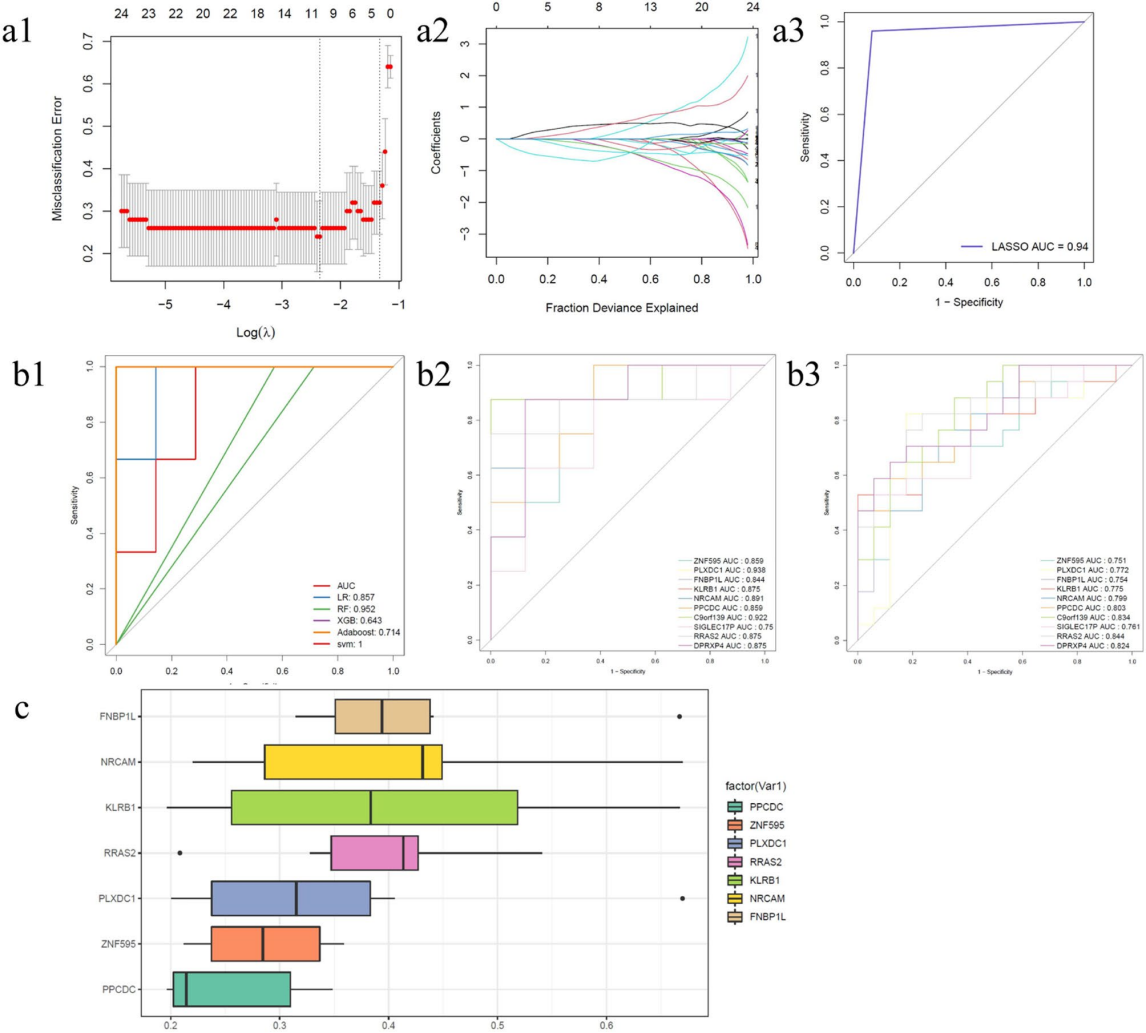
(**a**) Cross-validated LASSO model plot: (**a1, a2**). In actual analysis, we hope to find the position with the smallest cross-validation error. In **a1**, the dotted line is the position with the smallest cross-validation error. According to this position (λ.min), the corresponding horizontal axis log (λ) is determined. The number of characteristic genes is shown above. After finding the optimal log (λ) value, the corresponding gene and its coefficient are found in **a2**, as well as the proportion of residuals explained by the model; (**a3**) ROC curve for LASSO screening genes; (**b**) Machine Learning Model ROC Curves: (**b1**) ROC curves of five machine learning models; (**b2**) ROC curve of HUBgenes in GSE124272; (**b3**) ROC curve of HUBgenes in GSE150408; (**c**) HUBgene functional similarity analysis.

There were 10 HUBgenes (ZNF595, PLXDC1, FNBP1L, KLRB1, NRCAM, PPCDC, C9orf139, SIGLEC17P, RRAS2 and DPRXP4) screened out by LASSO (Fig. 5a). Through the ROC curves, it was clearly found that 10 HUBgenes had favorable reliability (Fig. 5b). Furthermore, it was found that 7 HUBgenes (ZNF595, PLXDC1, FNBP1L, KLRB1, NRCAM, PPCDC and RRAS2) have significant functional similarity (Fig. 5c).

### GSEA of HUBgenes

**Fig. 6:**
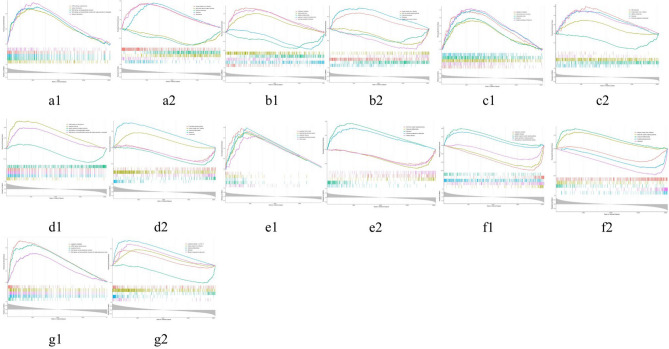
GSEA enrichment analysis of HUBgenes: (**a1**) ZNF595.GSEA.GO; (**a2**) ZNF595.GSEA.KEGG; (**b1**) PLXDC1.GSEA.GO; (**b2**) PLXDC1.GSEA.KEGG; (**c1**) FNBP1L.GSEA.GO; (**c2**) FNBP1L.GSEA.KEGG; (**d1**) KLRB1.GSEA.GO; (**d2**) KLRB1.GSEA.KEGG; (**e1**) NRCAM.GSEA.GO; (**e2**) NRCAM.GSEA.KEGG; (**f1**) PPCDC.GSEA.GO; (**f2**) PPCDC.GSEA.KEGG; (**g1**) RRAS2.GSEA.GO; (**g2**) RRAS2.GSEA.KEGG.

In the GO enrichment analysis, ZNF595 was mainly enriched in telomere maintenance, RNA splicing, via transesterification reactions and nuclear chromosome. PLXDC1 was involved in cytoplasmic translation, mitochondrial matrix and olfactory receptor activity. In addition, FNBP1L was mainly participated in cytoplasmic translation, mitochondrial inner membrane and mitochondrial matrix. KLRB1 was associated with mRNA splicing, via spliceosome, organellar ribosome and positive regulation of cytokine production. NRCAM was mainly enriched in maturation of LSU-rRNA, organellar large ribosomal subunit and organellar ribosome. PPCDC and RRAS2 took part in cytoplasmic translation, mRNA splicing, via transesterification reactions and organellar ribosome. And in the KEGG enrichment analysis, ZNF595 was mainly enriched in neuroactive ligand-receptor interaction, ribosome and spliceosome. PLXDC1 mainly participated in NOD-like receptor signaling pathway and ribosome. FNBP1L was mainly involved in DNA replication, ribosome biogenesis in eukaryotes and ribosome. KLRB1 and RRAS2 were associated with osteoclast differentiation and ribosome. NRCAM and PPCDC took part in NOD-like receptor signaling pathway, osteoclast differentiation and ribosome (Fig. 6a–g, Supplementary Data).

### Immune infiltration and cytokine analysis of HUBgenes

**Fig. 7:**
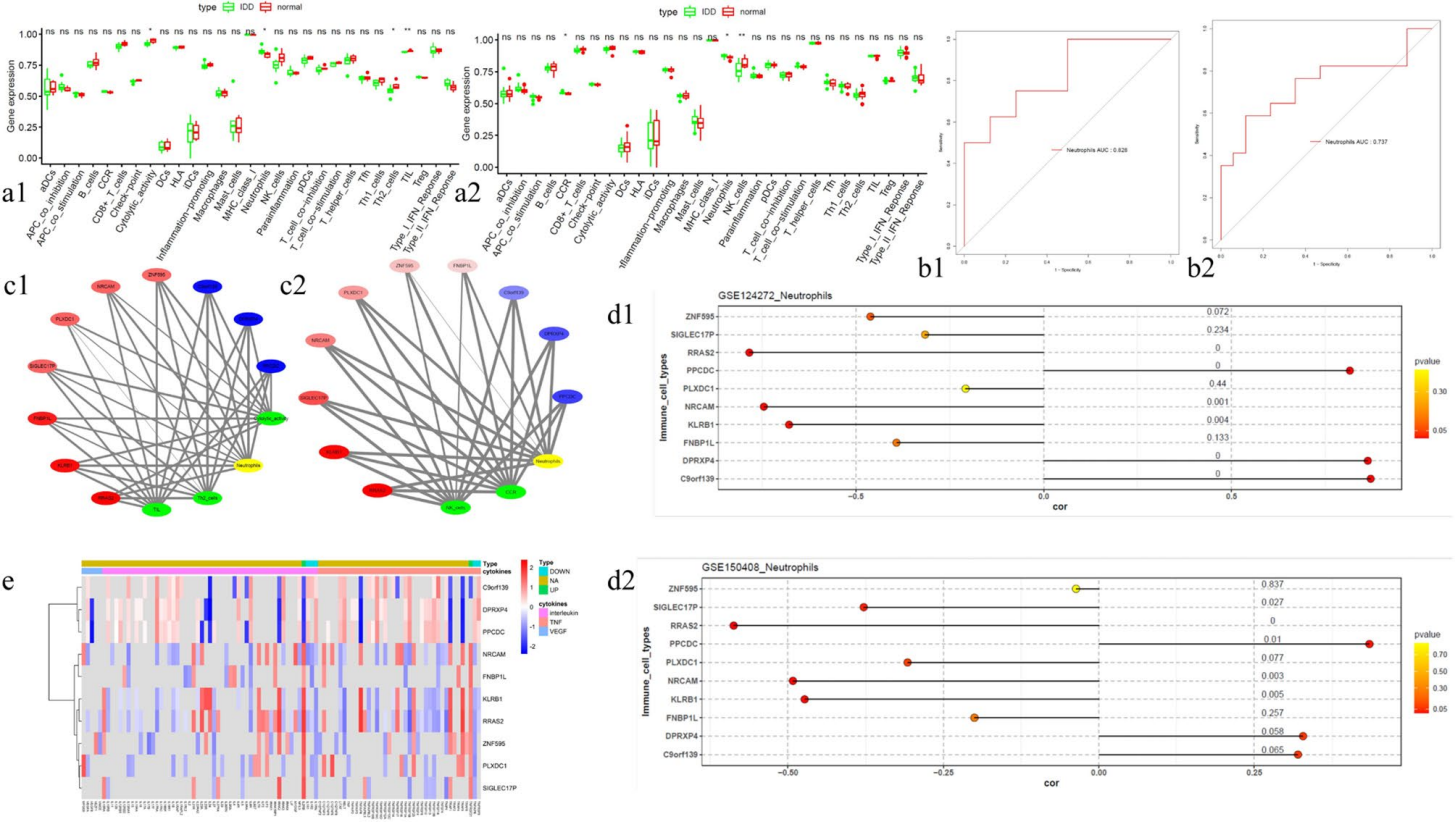
(**a**) Box plots of 29 immune gene sets in GSE124272 and GSE150408. **a1** and **a2** are box plots of 29 immune gene set contents in the IDD and normal groups of GSE124272 and GSE150408, respectively. (* p < 0.05, ** p < 0.01, *** p < 0.001); (**b**) ROC curves of key immune cells. **b1** and **b2** are the ROC curves of key immune cells in GSE124272 and GSE150408 of IDD and normal groups, respectively; (**c**) HUBgenes correlation network with key immune cells. The red nodes represent up-regulated genes, the blue represent down-regulated, the green represent differential immune cells, the yellow represent immune cells with significant differences in both datasets, and the thickness of the connection represents the magnitude of the correlation; (**d**) Lollipop charts of HUBgenes’ correlation with key immune cells. **d1** and **d2** are lollipop charts of the correlation between HUBgenes and key immune cells in GSE124272 and GSE150408 of IDD and normal groups, respectively; (**e**) Heat map of the correlation between HUBgenes and common cytokines.

Subsequently, neutrophil was screened out as key differential immune cell (Fig. 7a). The key differential immune cell that screened out had certain accuracy (Fig. 7b). In the correlation network, it was found that neutrophil related to all HUBgenes in two datasets (Fig. 7c). PPCDC, DPRXP4 and C9orf139 were positively correlated with neutrophil in two datasets, while ZNF595, SIGLEC17P, RRAS2, NRCAM, PLXDC1, FNBP1L and KLRB1 had a negative correlation (Fig. 7d). The significant correlation between HUBgenes and common cytokines were visualized by the heatmap (Fig. 7e).

### The construction of nomogram

**Fig. 8:**
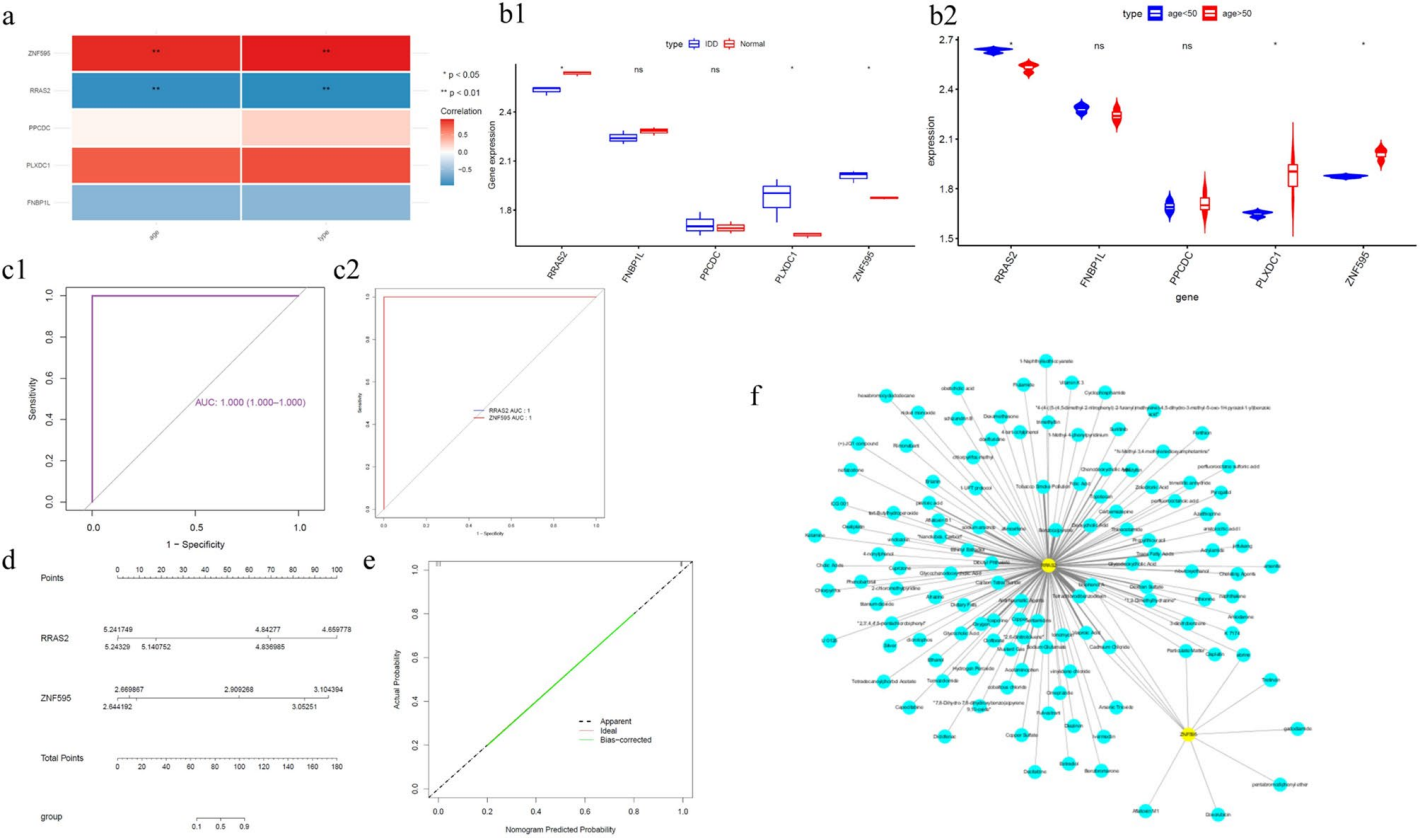
(**a**) Heatmap of correlation between significantly different HUBgenes and clinical features obtained in GSE167199; (**b1**) Expression box plot of HUBgenes in GSE167199; (**b2**) Expression box plot of HUBgenes in different age groups in GSE167199; (**c1**) SVM validation ROC curve in GSE167199; (**c2**) Validation ROC curve of HUBgenes in GSE167199; (**d**) Patient HUBgenes Nomogram; (**e**) Patient HUBgenes calibration curve; (**f**) Gene-drug network of HUBgenes. The blue nodes are drugs or small molecule compounds targeting HUBgenes, and the yellow are verified HUBgenes.

The correlation between HUBgenes (ZNF595, RRAS2, PPCDC, PLXDC1 and FNBP1L) and clinical features in the GSE167199 validation set were shown by heatmap (5 HUBgenes were not matched in GSE167199 validation set) (Fig. 8a). RRAS2 and ZNF595 were significantly different in the GSE167199 validation set, and they were also significantly related to clinical features (Fig. 8b). ROC curves also showed that two HUBgenes had favorable reliability (Fig. 8c). A nomogram was constructed based on RRAS2 and ZNF595 (Fig. 8d). The calibration curve was plotted based on the above nomogram, and it indicated that the prediction ability of the model was excellent (Fig. 8e). We obtained a total of 123 drugs According to the CTDbase. Among them, RRAS2 predicted 118 drugs such as Tetrachlorodibenzodioxin, bisphenol A and Benzo(a)pyrene. There were also 11 drugs (abrine, Aflatoxin M1, bisphenol A, etc.) based on ZNF595 (Fig. 8f, Supplementary Data).

### QuantitativeReal-timePCR (qPCR) identification

**Fig. 9:**
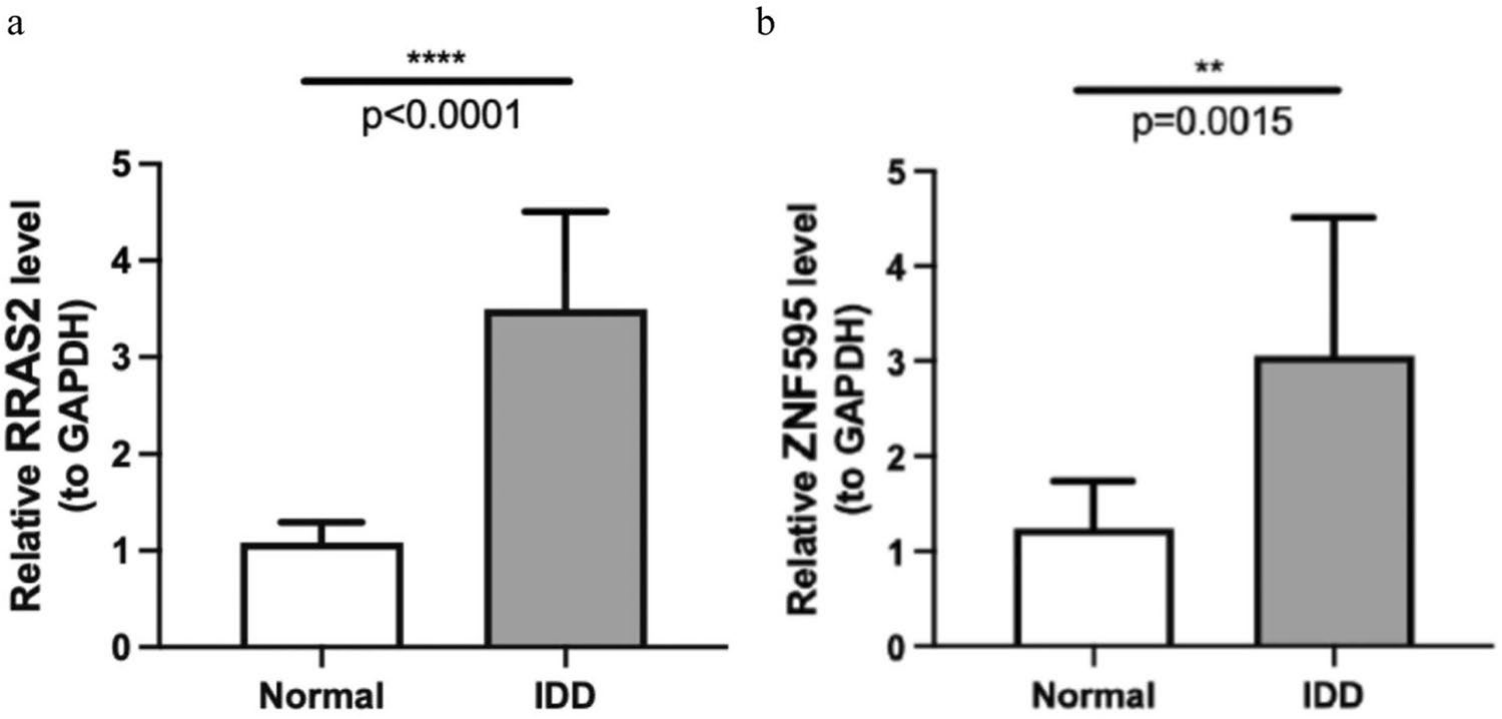
Validation of the expression of HUBgenes: (**a**) qRT-PCR detection of RRAS2 expression levels; (**b**) qRT-PCR detection of ZNF595 expression levels.

It can be seen that both RRAS2 and ZNF595 were significantly upregulated in IDD, which is not absolutely consistent with our above analyses results (Fig. 9, Table 2).

**Table 2:** Experiment results of qRT-PCR validation of HUBgenes.

Gene	Normal	IDD	p. value
RRAS2	1.0835 ± 0.2084	3.4981 ± 1.0100	< 0.0001
ZNF595	1.2432 ± 0.4965	3.0572 ± 1.4523	0.0015

## Discussion

Degeneration of IVDs is a major contributor to back, neck and radicular pain, which has already caused large socio-economic impact in the whole world^10^. Despite its universality, the initiation and progression of IDD are still not well understood, and a generic disease prediction model is still lacking^57^. In recent years, many clinical studies have indicated an association of iron overload with IDD’s incidence and pathological progression, which can be considered as an independent risk factor. Through an iron overload mouse model, researchers have observed that it promoted IDD and cartilage endplate degeneration in a dose dependent manner^22^. Moreover, a high dose of excess iron promoted chondrocytes ferroptosis^22^. Thus, iron overload is strongly related with the onset and development of IDD via ferroptosis, inhibiting ferroptosis could therefore be a promising therapeutic strategy for IDD induced by iron overload. As the most abundant internal modification of mRNA in eukaryotes, the relationship between m6A and ferroptosis in a variety of diseases has been an extremely hot research direction. It has already been proved that m6A-ferroptosis genes play important roles in diseases such as osteoporosis, acute kidney injury, cancers and so on^58–60^. Therefore, we have brought the question that how m6A-ferroptosis genes act their roles in IDD into the present study, aiming to identify m6A-ferroptosis genes in IDD and further hope to reveal the immune characteristics.

The research suggested that 7 HUBgenes (ZNF595, PLXDC1, FNBP1L, KLRB1, NRCAM, PPCDC and RRAS2) have significant functional similarity. ZNF595 was found to be methylation status associated with ambient temperature, which influences human epigenetic modifications^61^. It indicates that whether temperature is an influential factor in the mechanism of IDD might be a research direction in the future. While ZNF595 has already been found crucial in human gastric cancer and Dengue, its role in IDD remains unclear^62,63^. Our results suggested that the expression of ZNF595 is related to age, one of the high-risk factors for IDD. Whether its post-transcriptional regulation via m6A might contribute to IDD in relation to age or not is a research direction worth exploring.

PLXDC1 was suggested that it may regulate the biological functions of lipid metabolism and transport-related proteins through a series of biological processes, which is also significant in progression of IDD^64^. Therefore, it has potential to be a prognostic marker of IDD as of gastric cancer and colon cancer^65,66^. Moreover, the expression of PLXDC1 is significantly up-regulated in IDD group and down-regulated in older group, suggesting that it can be applied as therapeutic target in IDD via regulation of aging-related pathways. FNBP1L was predicted to function as a scaffold protein for microtubule, Rho family proteins, Formin-homology proteins and WAPS family proteins^67^. Researchers should study its role in IDD along the direction. Recent research found that expressions of favorably prognostic genes, including KLRB1 (encoding CD161), largely reflect tumor-associated leukocytes, indicating that KLRB1 could be a great break point for the following studies to elucidate the effects of IDD on body immunity^68^. NRCAM has already been found functioning in multiple diseases, such as liver cancer, Alzheimer’s disease, thyroid carcinoma and so on^69–71^. PPCDC is highly related to breast cancer^72^. However, there is still a lack of research on the mechanism of NRCAM and PPCDC in IDD.

As we all know, the incidence of IDD is mostly concentrated in middle-aged and elderly people, which increases in prevalence with age^3^. Cellular senescence has been viewed as a contributor to individual aging. In our results, the expression of RRAS2 was higher in normal group and younger group in comparative to IDD group and older group. Therefore, RRAS2 might be considered as a protective gene in IDD. RRAS2 preferred longer 3’ UTR usage and exhibited decreased expression in senescent cells^73^. Therefore, it was suggested that depletion of RRAS2 promoted senescence, while rescue of RRAS2 reversed senescence-associated phenotypes. It suggested that although RRAS2 has not been reported in any degeneration diseases, it could have important functions in the IDD prediction and treatment. However, much other research also reported that RRAS2 was overexpressed in various cancers, indicating its carcinogenic potential^74–76^. Thus, promoting the anti-aging mechanism of RRAS2 without leading to cancers is a research direction worth exploring as well.

We have also noticed that in the KEGG functional enrichment analysis, m6A-FRGs-DEGs were also involved in leishmaniasis and malaria. Although it may be in relation to environmental exposure or characteristics of the GEO datasets used in the present paper, it has been proposed that the association of m6A-FRGs-DEGs with leishmaniasis and malaria pathways is essentially the intersection of host epitranscriptome regulation and pathogen immune escape strategies^77–79^.

As for the experimental validation results, there were several factors that may cause the situation as we consider. First of all, our selection of the test samples is one of the most significant influence factors. For ethical and practical reasons, it was extremely difficult to obtain normal IVDs of young people, which has led to insufficient sample heterogeneity. Moreover, our samples were all from Chinese patients and mostly female, which were not consistent with the data from the GEO. Different human races and genders were other important reasons that may bring opposite gene expression results in our experiment. Last but not least, although we have already included 20 samples in our validation test, there were still not enough samples to prove our HUBgenes practically. In order to explore the expression and function of the selected genes in our study, future experiments using statistically large numbers of IVDs, applying multiple technical methods for verification, and conducting in-depth exploration of the specific mechanism of action of RRAS2 and ZNF595 in IDD will be definitely necessary. Besides, short-read sequencing platforms applied in our study might not fully capture repetitive elements of genes, it would be very helpful to use long-read sequencing technologies in future work.

## Conclusion

The focus of the study is to select a set of genes for further research on IDD. Through bioinformatic technologies, our research indicates that 10 HUBgenes (ZNF595, PLXDC1, FNBP1L, KLRB1, NRCAM, PPCDC, C9orf139, SIGLEC17P, RRAS2 and DPRXP4) are significantly associated with IDD, providing more evidence about the vital role of ZNF595 and RRAS2 in IDD. RRAS2 and ZNF595, which are genes highly differently expressed in IDD, on which our prediction model of IDD based. Further experimental mechanistic studies and clinical application studies are still necessary, and we will continuously focus on the role of HUBgenes in our research. We hope our work will provide new ideas for IDD diagnosis and management in the near future.

## Supplementary Information

Supplementary Information.

## Data Availability

Data is provided within the manuscript or supplementary information files.

## Abbreviations

SYSU: Sun Yat-Sen University
IDD: Intervertebral disc degeneration
m6A: N6-methyladenosine
FRGs: Ferroptosis-related genes
WGCNA: Weighted gene co-expression network analysis
DEGs: Differentially expressed genes
m6A-FRGs-DEGs: DEGs of m6A-FRGs related module genes
LASSO: Least absolute shrinkage and selection operator
GSEA: Gene set enrichment analysis
ssGSEA: Single sample gene set enrichment analysis
CTDbase: The comparative toxicogenomics database
qRT-PCR: The quantitative real-time fluorescence PCR
MSKs: Musculoskeletal disorders
IVD: Intervertebral disc
NPC: Nucleus pulposus cell
ECM: Extracellular matrix
AF: Annulus fibrosus
CEPs: Cartilaginous endplates
PCD: Programmed cell death
ROS: Reactive oxygen species
DNA: Deoxyribonucleic acid
RNA: Ribonucleic acid
AP-1: Activator protein-1
rRNA: Ribosomal ribonucleic acid
snRNA: Small nuclear ribonucleic acid
3’-UTR: 3’-Untranslated regions
GEO: Gene Expression omnibus
PCA: Principal component analysis
GO: Gene ontology
KEGG: Kyoto encyclopedia of genes and genomes
PPI: Protein–protein interaction
LR: Logistic regression
RF: Random forest
XGBoost: EXtreme gradient boosting
Adaboost: Adaptive boosting
SVM: Support vector machine
ROC: Receiver operating characteristic
PCR: Polymerase chain reaction
NK: Natural killer
